# Transcriptome and de novo analysis of *Rosa xanthina* f. *spontanea* in response to cold stress

**DOI:** 10.1186/s12870-021-03246-5

**Published:** 2021-10-15

**Authors:** Defeng Zhuang, Ce Ma, Li Xue, Zhen Li, Cheng Wang, Jiajun Lei, Xingfu Yuan

**Affiliations:** 1 Liaoning Academy of Agricultural Sciences, Shenyang, 110161 Liaoning China; 2 Agricultural College, Inner Mongolia Minzu University, Tongliao, 028000 China; 3 College of Horticulture, Shenyang Agricultural University, Shenyang, 110866 Liaoning China; 4 College of Life Sciences and Food Engineering, Inner Mongolia Minzu University, Tongliao, 028000 China

**Keywords:** Rose, Low-temperature stress, DEGs, Metabolic pathway

## Abstract

**Background:**

Rose is one of most popular ornamental plants worldwide and is of high economic value and great cultural importance. However, cold damage restricts its planting application in cold areas. To elucidate the metabolic response of rose under low temperature stress, we conducted transcriptome and de novo analysis of *Rosa xanthina* f. *spontanea*.

**Results:**

A total of 124,106 unigenes from 9 libraries were generated by de novo assembly, with N50 length was 1470 bp, under 4 °C and − 20 °C stress (23 °C was used as a control). Functional annotation and prediction analyses identified 55,084 unigenes, and 67.72% of these unigenes had significant similarity (BLAST, E ≤ 10^− 5^) to those in the public databases. A total of 3031 genes were upregulated and 3891 were downregulated at 4 °C compared with 23 °C, and 867 genes were upregulated and 1763 were downregulated at − 20 °C compared with 23 °C. A total of 468 common DEGs were detected under cold stress, and the matched DEGs were involved in three functional categories: biological process (58.45%), cellular component (11.27%) and molecular function (30.28%). Based on KEGG functional annotations, four pathways were significantly enriched: metabolic pathway, response to plant pathogen interaction (32 genes); starch and sucrose metabolism (21 genes); circadian rhythm plant (8 genes); and photosynthesis antenna proteins (7 genes).

**Conclusions:**

Our study is the first to report the response to cold stress at the transcriptome level in *R. xanthina* f. *spontanea*. The results can help to elucidate the molecular mechanism of cold resistance in rose and provide new insights and candidate genes for genetically enhancing cold stress tolerance.

**Supplementary Information:**

The online version contains supplementary material available at 10.1186/s12870-021-03246-5.

## Background

Rose is one of the most common ornamental plants, has high economic value, and is very popular with people worldwide. However, due to a lack of cold tolerance, its planting application is affected by low temperatures in cold regions. Cold resistance in woody plants is a complex metabolic process. Generally, plant growth and development stop when winter arrives, and cold tolerance and dormancy gradually form [1]. In addition, this process will result in variation in morphological traits at the transcriptional/biochemical level that then improve the stability of the membrane system, which can survive safely in winter, such as in most temperate woody plants [2].

A transcriptome represents the sum of all RNA that is transcribed in a functional state in a specific tissue or cell at a certain stage; it mainly includes mRNA and noncoding RNA [3]. Expression of the whole genome was revealed at the whole transcriptional level under abiotic stress to identify transcripts related to cold tolerance. It is of great significance to construct the transcriptional regulatory network of the genome under abiotic stress genome in terms of the complex regulatory network involved in increasing abiotic stress adaptation and tolerance [4, 5]. Transcriptome sequencing has already been performed for plants under low-temperature stress, such as in *Camellia sinensis* and *Populus euphratica* [6, 7].

Recently, there have been many studies on the transcriptome in rose. Abundant genetic information has been obtained on resistance and development with respect to transcriptome sequencing by using roots, leaves, flowers and fruits of rose as materials [8–11]. The fusion strategy combining the genome and proteome can provide a certain theoretical basis for resolving the biological problems of rose in the future. Transcriptome sequencing analysis has been used to study cold stress in the leaves in *R. multiflora* [12], fruit in blueberry [13] and floral buds in *Rosa hybrida* [14]. To date, transcriptomic information in rose has not been clarified because of the complexity of cold resistance mechanisms.

*Rosa xanthina,* a wild species of Sect. *Pimpinellifoliae,* is native to northeastern and northern China. In addition, *R. xanthina* f. *spontanea* has high cold/drought tolerance and disease resistance and is an important germplasm resource in the breeding of modern rose [15]. In the present study, we carried out transcriptome sequencing analysis of *R. xanthina* f. *spontanea* under low-temperature stress to clarify the functions and metabolic pathways associated with DEGs, which can provide a theoretical foundation for the cold-resistance mechanism in rose.

## Results

### Transcriptome sequencing and assembly

The original data obtained by sequencing with an Illumina HiSeq 4000 were transformed into raw reads by base calling. The total number of nucleotides obtained from nine libraries was 64.52 G, and the total number of nucleotides was between 5.23 G and 7.59 G in each sample. The Q30 ratio of each sample was greater than 94%, and the GC content was relatively consistent, at approximately 47% (Table S1). A total of 124,106 transcripts and 55,084 nonredundant unigenes were obtained from nine libraries. The average length of the unigenes was 661 bp, and the N50 length was 1470 bp. Consequently, the sequencing data quality was high and met the requirements for subsequent analysis. There were 29,582 unigenes with lengths of 200–500 bp, accounting for 53.70%; 10,103 unigenes with lengths of 500–1000 bp, accounting for 18.34%; 10,112 unigenes ranging from 1000 to 2000 bp, accounting for 18.35%; and 5287 unigenes with lengths greater than 2000 bp, accounting for 9.6% (Table S2).

### Functional annotation of unigenes

The Nr, Swiss-Prot (a manually annotated and reviewed protein sequence database), Pfam (protein family), KOG (Clusters of Orthologous Groups of proteins), KEGG and GO databases were used to annotate all unigenes with comprehensive gene function information. In the present study, a total of 37,303 unigenes were successfully annotated in the *R. xanthina* f. *spontanea* database, representing 67.72% of all unigenes (55,084). Furthermore, *Fragaria_vesca* presented the highest frequency in the annotation results, with a total of 19,964 comments, accounting for 53.52% of all the sequences, followed by *Nelumbo nucifera* (9.0%), *Prunus persica* (3.27%), *Phaseolus vulgaris* (3.13%), *Vitis vinifera* (2.78%) and *Prunus mume* (2.36%) (Fig. S1; Table S3).

A total of 31,258 (56.75%) unigenes were assigned to GO terms in the cellular component, molecular function and biological process categories; these unigenes were further classified into 50 GO terms (Fig. S2).

Within the cellular component category, a total of 22,417 DEGs were assigned under 4 °C and − 20 °C stress (23 °C as the control), which indicated that the union of all the DEGs was mainly related to the nucleus, cytoplasm, integral component of membrane and chloroplast. For the molecular function, most of the DEGs were enriched for molecular function, protein binding and ATP binding. In the biological process category, biological process, regulation of transcription and DNA–template were the most enriched (Table S4).

To identify the metabolic pathways involved in cold stress of *R. xanthina* f. *spontanea* leaves, 21,992 DEGs were mapped to the KEGG database, and the mainly 19 different KEGG pathways are assigned in Fig. S3 (Table S5). Among these pathways, carbohydrate metabolism (2596, 11.80%), translation (2325, 10.57%) and folding, sorting and degradation (2036, 9.26%) were the most extensively overrepresented pathways. In addition, metabolic pathways related to environmental adaptation, transport and catabolism were also found. In summary, amino acid metabolism, translation and signal transduction are involved in almost every aspect of plant life. Furthermore, it was demonstrated that highly informative and wide coverage was obtained from transcriptome sequencing of leaves in *R. xanthina* f. *spontanea* and can be used to analyse the gene products of metabolic pathways and information processing pathways at the molecular level.

### DEGs in response to low-temperature stress

**Fig. 1:**
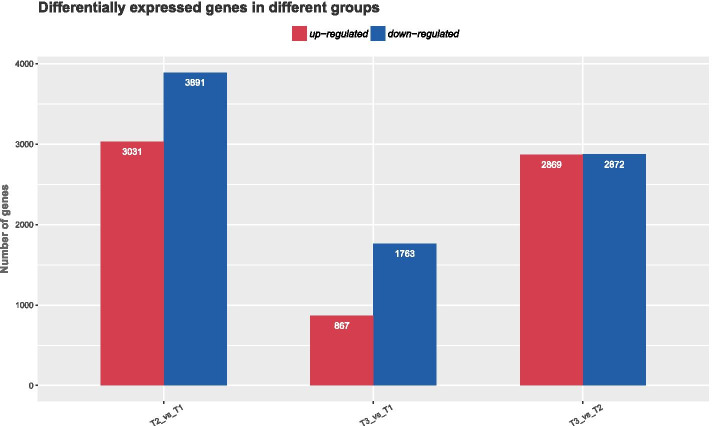
The column and Venn diagrams of DEGs assembled under low-temperature stress across three sets of comparisons expressed as 4 °C vs 23 °C (control), − 20 °C vs 23 °C and 4 °C vs − 20 °C, respectively (*P* < 0.05)

In this study, differentially expressed genes were used for comparative analyses of tolerance to low temperatures (4 °C(T2), − 20 °C(T3), 23 °C (T1, control), Fig. 1). A total of 6922 genes were identified at 4 °C; 3031 genes were upregulated, and 3891 were downregulated. In the case of − 20 °C, 2630 DEGs were detected, including 867 upregulated genes and 1763 downregulated genes. By comparing the − 20 °C and 4 °C treatments, 5741 DEGs were identified; 2869 genes were upregulated and 2872 genes were downregulated. All DEGs were less abundant at − 20 °C than at 4 °C regardless of whether they were upregulated or downregulated. According to the Venn diagram, 468 DEGs were commonly involved in the above three groups, suggesting that these genes may play an important role in cold tolerance in *R. xanthina* f. *spontanea*.

### Gene ontology enrichment analyses of DEGs

We elucidated the GO terms related to the biological functions of the DEGs that were significantly altered under the three treatments (23 °C, 4 °C and − 20 °C). The significantly enriched GO terms (*p <* 0.05) were identified, and classification of the GO terms was performed according to the NCBI nonredundant (NR) annotation using Blast2 GO software. The DEGs were defined as enriched for GO terms when the above conditions were met. A total of 11,705 unigenes were successfully assigned to at least one GO term. All the GO terms were classified into functional groups, including three main categories: biological processes, cellular components and molecular functions. In total, 206 significantly enriched GO terms, including 6853 unigenes, at 4 °C were identified. In the biological process category, there were 69 GO terms (2001). For cellular components, there were 22 GO terms (1734). Within the molecular function category, there were 115 GO terms (3118). The top three most enriched GO terms were response to chitin (52), chloroplast (782), and transcription factor activity and sequence-specific DNA binding (291). Moreover, the results showed that the most enriched GO term was biological process (789) (Table S6).

In comparison, 219 significantly enriched GO terms, including 4708 unigenes, were found at − 20 °C. In the biological process category, there were 128 GO terms (2073). For cellular components, there were 19 GO terms (1150). Within the molecular function category, there were 72 GO terms (1485). The top three most enriched GO terms were transcription factor activity and sequence-specific DNA binding, response to chitin, and defence response to fungus. Moreover, the results showed that the most enriched GO term was plasma membrane (384 genes), and the second was integral component of membrane (327 genes) (Table S7), suggesting that the genes in these processes may play important roles in low temperature perception.

### KEGG enrichment analysis of DEGs

The significantly enriched KEGG metabolic pathways associated with the DEGs in *R. xanthina* f. *spontanea* were analysed (*p*<0.05). Under 4 °C treatment, 1014 DEGs were assigned sixteen pathways (Table 1); 321 genes were upregulated and 693 were downregulated. For the − 20 °C treatment, 647 DEGs were enriched in 20 pathways; 218 genes were upregulated and 429 genes were downregulated. A total of nine common metabolic pathways were annotated, such as starch and sucrose metabolism and plant-pathogen interactions, and these pathways likely play important roles in low-temperature perception during cold stress treatment. Additionally, the DEGs related to photosynthesis-antenna proteins and circadian rhythm-plant pathways showed that there were more upregulated genes than downregulated genes, and the other pathways showed more downregulated genes than upregulated genes. Under 4 °C treatment, seven pathways (phenylpropanoid biosynthesis, ribosome biogenesis in eukaryotes, etc.) showed more downregulated genes than upregulated genes. In comparison, the eleven most significantly enriched pathways (plant hormone signal transduction, amino sugar and nucleotide sugar metabolism, etc.) are listed in Table 1 for − 20 °C, and more upregulated genes than downregulated genes were involved in the mapped pathways for monoterpenoid biosynthesis and tryptophan metabolism (Table S8, S9).

**Table 1:** Summary of KEGG pathway functional annotations under cold stress treatment

Pathway	ID code	4 °C vs. 23 °C				−20 °C vs. 23 °C			
		Total number of DEGs	Up	Down	*P* Value	Total number of DEGs	Up	Down	*P* Value
Starch and sucrose metabolism	ko00500	221	37	184	1.36E-08	75	22	53	0.036091
Photosynthesis - antenna proteins	ko00196	21	18	3	0.005443	12	11	1	0.002156
Circadian rhythm - plant	ko04712	48	31	17	0.016043	37	22	15	3.92E-08
Plant-pathogen interaction	ko04626	274	103	171	0.017747	176	42	134	6.66E-16
Glycerolipid metabolism	ko00561	57	22	35	0.025967	29	12	17	0.004703
Nitrogen metabolism	ko00910	26	7	19	0.027501	15	0	15	0.003943
Anthocyanin biosynthesis	ko00942	12	4	8	0.040398	9	3	6	0.001390
Flavonoid biosynthesis	ko00941	28	5	23	0.045727	20	7	13	0.000119
Zeatin biosynthesis	ko00908	20	4	16	0.045810	14	8	6	0.000760
Fatty acid elongation	ko00062	28	4	24	0.002586	–	–	–	–
Carotenoid biosynthesis	ko00906	32	13	19	0.003827	–	–	–	–
Phenylpropanoid biosynthesis	ko00940	84	28	56	0.004435	–	–	–	–
Base excision repair	ko03410	30	6	24	0.006088	–	–	–	–
Ribosome biogenesis in eukaryotes	ko03008	75	25	50	0.014468	–	–	–	–
Ascorbate and aldarate metabolism	ko00053	49	13	36	0.025578	–	–	–	–
Riboflavin metabolism	ko00740	9	1	8	0.028072	–	–	–	–
Plant hormone signal transduction	ko04075	–	–	–	–	102	38	64	4.96E-07
Amino sugar and nucleotide sugar metabolism	Ko00520	–	–	–	–	60	14	46	0.000918
Cutin, suberine and wax biosynthesis	Ko00073	–	–	–	–	14	5	9	0.001240
Caffeine metabolism	ko00232	–	–	–	–	6	3	3	0.010312
Stilbenoid, diarylheptanoid and gingerol biosynthesis	ko00945	–	–	–	–	13	5	8	0.015090
Monoterpenoid biosynthesis	ko00902	–	–	–	–	7	5	2	0.018729
Tryptophan metabolism	ko00380	–	–	–	–	17	11	6	0.029553
Isoquinoline alkaloid biosynthesis	ko00950	–	–	–	–	13	3	10	0.036862
Tropane, piperidine and pyridine alkaloid biosynthesis	ko00960	–	–	–	–	11	4	7	0.038950
Brassinosteroid biosynthesis	ko00905	–	–	–	–	9	2	7	0.039466
Sesquiterpenoid and triterpenoid biosynthesis	ko00909	–	–	–	–	8	1	7	0.049619
Total		1014	321	693		647	218	429	

### GO enrichment analysis of 468 DEGs

**Fig. 2:**
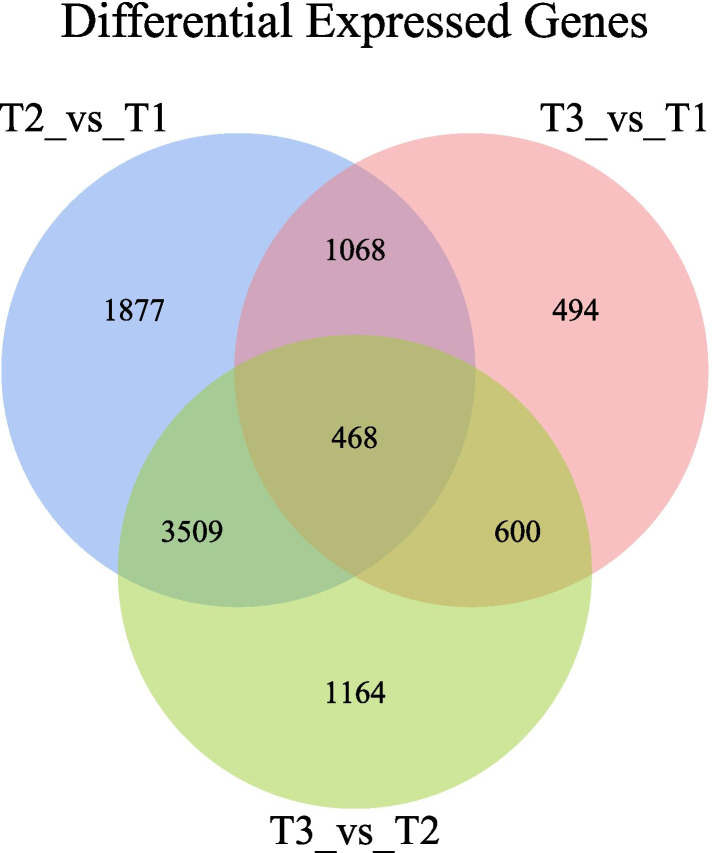
GO enrichment analysis of 468 DEGs. The unigenes were classified into three main categories: biological process, cellular component and molecular function

In this study, to reveal which biological functions were significantly related to the common DEGs we obtained, a GO functional enrichment analysis was carried out (*p*<0.05). The results indicated that the DEGs involved in biological processes, cellular components and molecular functions accounted for 58.45, 11.27, and 30.28% of the total DEGs, respectively. Consequently, most DEGs were significantly correlated with some biological functions. We found that the DEGs were classified into 93 biological processes, mainly focused on transcriptional regulation, DNA template; transcription, DNA template; response to chitin; response to abscisic acid; response to cold; etc. Regarding cellular components (16), the DEGs were involved in chloroplasts, extracellular regions, chloroplast thylakoid membranes, etc. With respect to molecular functions (43), the DEGs were mainly involved in molecular function, transcription factor activity, sequence-specific DNA binding, and sequence-specific DNA binding (Fig. 2; Table S10).

### KEGG pathway enrichment analysis of 468 DEGs

To more precisely investigate the variation in metabolic pathways in leaves during low-temperature stress, statistical pathway enrichment analysis on the DEGs was carried out using the KEGG database. A total of 293 DEGs under low-temperature stress were assigned to 85 different KEGG pathways. There were four significantly enriched pathways (*p* < 0.05): plant-pathogen interaction, starch and sucrose metabolism, plant circadian rhythm and photosynthesis-antenna proteins (Table 2; Table S11). When the temperature reached 4 °C and − 20 °C, most DEGs in the plant-pathogen interaction and starch and sucrose metabolism pathways were downregulated. In addition, the genes in the plant circadian rhythm pathway showed similar numbers of, downregulated and upregulated genes. However, seven DEGs in the photosynthesis-antenna protein pathway were upregulated.

**Table 2:** Summary of KEGG pathway functional annotations for 468 DEGs

Pathway	ID code	The number of DEGs in metabolic pathway	Number of 468 DEGs			
			4 °C vs. 23 °C		−20 °C vs. 23 °C	
			Up	down	Up	Down
Plant-pathogen interaction	ko04626	32	6	26	4	28
Starch and sucrose metabolism	ko00500	21	4	17	3	18
Circadian rhythm - plant	ko04712	8	3	5	4	4
Photosynthesis - antenna proteins	ko00196	7	7	0	7	0

### Plant-pathogen interaction metabolic pathway

In the metabolic pathway of plant-pathogen interactions, as shown in Table 2, after removing the genes with TPM values less than 10, we observed 6 upregulated genes and 22 downregulated genes at 4 °C and 4 upregulated genes and 24 downregulated genes at − 20 °C. The genes cyclic nucleotide-gated ion channel 1 (*CNGC1*, (*TRINITY_DN27111_c1_g1*, *TRINITY_DN28981_c1_g5*)), leucine-rich repeat receptor-like serine/threonine-protein kinase (*LRR-RLK*) and inactive receptor kinase (*At4g23740*) were significantly upregulated at 4 °C and − 20 °C, but the genes cyclic nucleotide-gated ion channel 1 (*CNGC1*, (*TRINITY_DN 32274_c2_g1*)) and ethylene-responsive transcription factor (*ABR1*) were upregulated at 4 °C but downregulated at − 20 °C. The others were all downregulated at 4 °C and − 20 °C, including WRKY transcription factors (*WRKY11*, *WRKY17*, *WRKY24*, *WRKY41*, *WRKY48*, *WRKY53*), calcium-binding proteins (*CML19*, 2 *CML27*, *CML37*), ethylene-responsive transcription factors (*ERF1A*, *ERF017*, *ERF020*, *ERF109*), and dehydration-responsive element-binding protein 1B (*DREB1D*) (Table 2;Table S11).

### Starch and sucrose metabolism pathway

Starch and sucrose metabolism included two processes: synthesis and decomposition. Six genes with TPM values less than 10 were removed, leaving 4 upregulated and 11 downregulated genes at 4 °C and 2 upregulated and 13 downregulated genes at − 20 °C (Table 2). In addition, the leucine-rich repeat receptor-like serine/threonine-protein kinase (*LRR-RLK*) and beta-glucosidase (*BGLU12*) genes regulating glucose synthesis were significantly upregulated at 4 °C, but the beta-glucosidase (*F26G, At4g27290*) genes were downregulated. The gene beta-amylase 1 (*BAM1*), which regulates maltose synthesis, was upregulated, but *BAM2* and *BAM3* were downregulated*.* Additionally, a TMV resistance protein was annotated to be upregulated in *Prunus mume*, indicating a key role of sucrose synthase. The other downregulated genes were galacturonosyl transferase (*GATL3*, *GATL10*), cellulose synthase A catalytic subunit 5 (*CESA2*, *CESA6*), 1,4-alpha-glucan-branching enzyme 1 (*SBE1*), isoamylase 1 (*ISA1*), and APO protein 1 (*APO1*). Beta-glucosidase (*At4g27290*) and *LRR-RLK* were upregulated at 4 °C but downregulated at − 20 °C (Table 2; Table S11).

### Circadian rhythm-plant metabolic pathway

In the circadian rhythm-plant metabolic pathway, 8 genes were significantly different among the three groups, including 3 upregulated and 5 downregulated genes at 4 °C and 4 upregulated and 4 downregulated genes at − 20 °C (Table 2). Removing the gene with a TPM value less than 10, among the remaining 7 genes, the *APRR5* and *GI* 2 genes were upregulated at 4 °C, and *APRR5* was also upregulated at − 20 °C, but its expression was lower at 4 °C. In addition, both *MYB23* and *C1* were significantly upregulated and annotated as trichome differentiation protein and transcription factor WER, respectively; the other genes were downregulated (Table 2; Table S11).

### Photosynthesis - antenna proteins metabolic pathway

Antenna proteins are the most important part of the light harvesting complex *(LHC*) in terms of light energy collection during photosynthesis. In the metabolic pathway of photosynthesis-antenna proteins, 7 genes were significantly upregulated among the three temperature groups. Gene annotation showed that *CAB2,* a hypothetical protein GLYMA was continuously upregulated under the 4 °C and − 20 °C treatments, and its TPM value was 10.64 at − 20 °C. The other six chlorophyll a-b binding protein genes (*LHCB3*, 2 *AB80*, 2 *CAB-151*, *CAB40*) showed high expression at 4 °C but slightly decreased expression at − 20 °C (Table 2; Table S11).

### qRT-PCR validation of the DEGs

**Fig. 3:**
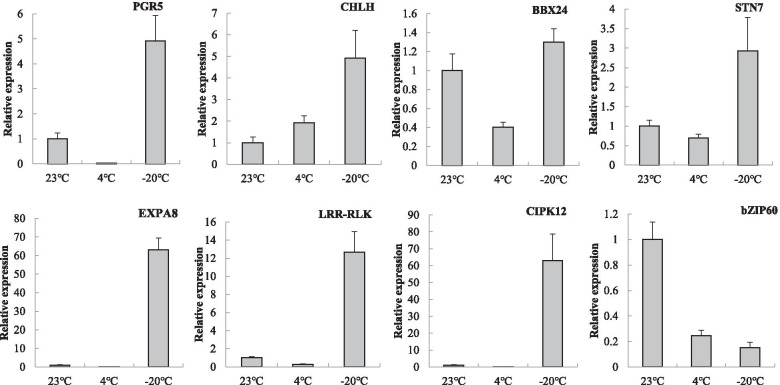
qRT-PCR analysis of expression levels of randomly selected genes in *R. xanthina* f. *spontanea* along with corresponding results under 4 °C, − 20 °C and 23 °C stress, respectively. Standard errors (SE) bars are shown within each of the columns

Based on the results of transcriptome annotation under low-temperature stress, eight DEGs related to cold resistance were screened (Fig. 3; Table S12). The fold change in expression was analysed by qRT-PCR before and after low-temperature stress. The results indicated that the expression of the *PGR5*, *CHLH*, *BBX24*, *STN7*, *EXPA8*, *LRR-RLK* and *CIPK12* unigenes was upregulated, and that of *bZIP60* was downregulated. The same trend was observed in the high-throughput sequencing analysis. Consequently, these genes mentioned above may be directly correlated with cold resistance in *R. xanthina* f. *spontanea*, and the results further confirmed the reliability of our transcriptome data.

## Discussion

In view of the lack of genome-wide data for nonmodel plants, high-throughput transcriptome sequencing technology can determine the sequences of each transcriptional fragment and rare transcript for any species. There is no need to understand the genetic information of that species. Thus, this method is crucial to the study nonmodel plants. In the present study, the transcriptional data we obtained were compared with data from known protein databases (Nr, Swiss-Prot, COG, KEGG) according to the principle that if the gene structure is similar, then the function is homologous. The functional genes were annotated with the support of a powerful bioinformatics platform. To date, this platform has been successfully used in previous reports on nonmodel plants [16–20] and provides abundant genetic data sources for plant functional genomics. High-throughput sequencing was performed on the leaves of *R. multiflora* under low-temperature stress using an Illumina HiSeq™ 4000, and a total of 55,084 unigenes were identified. Of these unigenes, 37,303 unigenes (67.7%) had functional annotations when compared with the Nr database, and the most frequent plant was *Fragaria vesca*, with 53.5% of the total unigenes (Fig. S1; Table S3). The results were consistent with those from previous studies [12, 21], where the unigenes represented 32.8% of *R. multiflora* and 64.6% of *R. beggeriana* Schrenk genes.

In the present study, there were more downregulated genes than upregulated genes at 4 °C and − 20 °C in *R. xanthina* f. *spontanea*. This result is similar with those of Kou et al. [22] in potato and Zhou et al. [23] in Chinese jujube, which indicated that different cultivars may have similar responses and cold resistance mechanisms. However, these results are not consistent with those of Niu et al. [24] in *Prunus persica* under freezing stress treatment, in which the number of upregulated genes was greater than that of downregulated genes. At the same time, these results are also different from those of Zhang et al. [12] in *R. multiflora*, which may be due to the use of different experimental materials. In addition, the results suggested that all the DEGs were more abundant under 4 °C stress than at − 20 °C regardless of whether they were upregulated and downregulated, suggesting that related genes may be expressed by cold signals in the early stage of low temperature stress in *R. xanthina* f. *spontanea*.

By performing KEGG pathway analyses, among all the DEGs of the three treatment groups, the commonly expressed genes were enriched in four pathways: plant-pathogen interaction, starch and sucrose metabolism, circadian rhythm-plant and photosynthesis-antenna proteins (Table 2). In *R. beggeriana,* many DEGs were enriched in starch and sucrose metabolism and the plant-pathogen interaction pathway at 4 °C [21]. In addition, Du et al. [25] performed transcriptome analysis under cold stress in *Brassica napus* L., and the results indicated that many DEGs were enriched in the circadian rhythm-plant, plant-pathogen interaction metabolic, plant hormone signal transduction and secondary metabolic pathways. Photosynthesis was the first metabolic process inhibited during chilling injury [26]. Chloroplasts are the main organelles affected by cold conditions [2].

In the present study, 7 light-harvesting complex II chlorophyll a/b binding protein genes enriched in the photosynthesis-antenna protein pathway were significantly upregulated, and the gene expression increased dramatically, especially in *CAB-151* and *CAB40*. Therefore, the genes encoding light-harvesting complex II chlorophyll a/b binding proteins were upregulated under cold stress in *R. xanthina* f. *spontanea*, improving photosynthesis and enhancing cold resistance. The final products of photosynthesis were starch and sucrose. Based on the starch and sucrose metabolism pathway, we found that many genes were upregulated or downregulated and were involved in complex metabolic reactions to adapt to low temperature stress. Of these genes, only the *LRR-RLK* gene of the leucine-rich repeat receptor-like serine/threonine-protein kinase family was upregulated at 4 °C and − 20 °C, indicating that the *LRR-RLK* gene may play an important role in regulating cold stress responses. Additionally, there was a circadian rhythm to the expression of *cab*, which may be controlled by the biological clock [27–29]; this was consistent with the pathways enriched in circadian rhythm plants. The timing function of the plant biological clock is related to the level of carbohydrates in plant cells, and carbohydrates are produced by photosynthesis in plants, which is a key metabolic input of the plant biological clock. Carbohydrates, as feedback substances that accumulate in plants, can regulate the timing and reset function of the plant biological clock to the external cycle [30]. Regarding GO enrichment analyses of the DEGs common to the three treatment groups, for cellular components, most DEGs were enriched in chloroplasts, extracellular regions, and chloroplast thylakoid membranes, and the results were consistent with the location of photosynthesis. In comparison to those at 4 °C, more DEGs were enriched in secondary metabolic pathways at − 20 °C, suggesting that cold signalling enhanced antenna protein genes during cold stress. Furthermore, to enhance photosynthesis, promote carbon metabolism, and strengthen cold resistance in *R. xanthina* f. *spontanea*, with a decrease in temperature, a number of genes that regulate metabolism were activated to protect against low temperature. In this study, the response mechanism under cold stress was systematically analysed at the transcriptional level in *R. xanthina* f. *spontanea* to elucidate cold-related metabolic pathways and lay a foundation for exploring the key functional genes associated with cold tolerance. Therefore, it is of great significance to accelerate the progress of genetic improvement of cold tolerance in rose.

## Conclusions

Our study is the first to report on the response to cold stress at the transcriptome level in *R. xanthina* f. *spontanea*, which can provide a theoretical basis for further studies on the molecular mechanism of cold resistance in rose. Important genes involved in plant-pathogen interactions, starch and sucrose metabolism, circadian rhythm-plant and photosynthesis-antenna proteins were significantly enriched under low-temperature stress, which most of the genes were downregulated. In comparison to 4 °C, secondary metabolites play an important role at − 20 °C. The results of this study may be beneficial for further studies on cold tolerance mechanisms in rose and other plants.

## Methods

### Plant materials and low-temperature treatment

The plant materials of *R. xanthina* f. *spontanea* used in our study were planted in Liaoning Research Institute of Cash Crops, Liaoning Academy of Agricultural Sciences, Liaoning, 110,161, China. It is necessary to obtaining permissions to collect these samples, Jiajun Lei professor (College of Horticulture, Shenyang Agricultural University, Liaoning 110,866, China) undertook the formal identification of the samples, and its detailed information was described based on Help Me Find (https://www.helpmefind.com/gardening/l.php?l=2.46960.1). Cutting propagation was performed in the greenhouse; annual cutting seedlings with the same growth vigour and management conditions were placed in a variable temperature (23 °C) climate chest 1 week prior (16 h light, 8 h darkness and 70% relative humidity), and the temperature was dropped 2 °C per hour to 4 °C and − 20 °C. This temperature was maintained for 12 h and then heated to 23 °C at 2 °C per hour. The leaf blades were quickly placed in liquid nitrogen and stored at − 80 °C. All the experiments were repeated three times.

### RNA extraction, library construction and sequencing

Total RNA was extracted using TRIzol reagent (Invitrogen, CA, USA) following the manufacturer’s procedure. The total RNA quantity and purity were analysed by a Bioanalyzer 2100 and RNA 6000 Nano LabChip Kit (Agilent, CA, USA) with a RIN number > 7.0. Approximately 10 μg of total RNA representing a specific adipose type was subjected to isolate poly(A) mRNA purification with poly-T oligo-attached magnetic beads (Invitrogen, CA, USA). Following purification, the poly(A) or poly(A) + RNA fraction was fragmented into small pieces using divalent cations under elevated temperature. Then, the cleaved RNA fragments were reverse-transcribed to create the final cDNA library in accordance with the protocol for the mRNA-Seq sample preparation kit (Illumina, San Diego, USA), and the average insert size for the paired-end libraries was 300 bp (±50 bp). Then, paired-end sequencing was performed by an Illumina HiSeq 4000 (LC-Bio, China) following the vendor’s recommended protocol. The raw sequence data have been submitted to the NCBI Short Read Archive with accession code PRJNA724822.

### De novo assembly, unigene annotation and differential expression analysis

First, de novo assembly, functional annotation and classification of the unigenes were performed, and cutadapt [31] and in-house Perl scripts were used to remove the reads that contained adaptor contamination, low-quality bases and undetermined bases. And, the reliability of the unigenes assembly was tested using BUSCO (ver. 5.1.2) (Fig. S4). Then, sequence quality was verified using FastQC (http://www.Bioinformatics.babraham.ac.uk/projects/fastqc/), including the Q20, Q30 and GC content of the clean data. All downstream analyses were based on clean data with high quality. De novo assembly of the transcriptome was performed with Trinity 2.4.0 [32]. Trinity groups transcripts into clusters based on shared sequence content.

All assembled unigenes were aligned against the NCBI nonredundant protein sequences (Nr) (http://www.ncbi.nlm.nih.gov/), GO (http://www.geneontology.org), SwissProt (http://www.expasy.ch/sprot/), KEGG (http://www.genome.jp/kegg/) and eggnog (http://eggnogdb.embl.de/) databases using DIAMOND [33] with a threshold of E value< 0.00001.

Differentially expressed unigene analysis based on Salmon [34] was used to determine the expression level for unigenes by calculating transcripts per million (TPM) [35]. The DEGs were selected with log2 (fold change) > 1 or log2 (fold change) < − 1 and with statistical significance (*p* < 0.05) by the R package edgeR [36]. Next, GO and KEGG enrichment analyses were repeated based on the DEGs identified by the OmicStudio tools at https://www.omicstudio.cn/tool/22.

### qRT-PCR validation

The leaves of *R. xanthina* f. *spontanea* were collected and treated at 23 °C, 4 °C and − 20 °C, and 10 DEGs selected at random were used for qRT-PCR validation. Total RNA was extracted by using a polysaccharide polyphenol Plant RNA Isolation Kit (N1005, Biobase Technologies Co., Ltd., ChengDu, China), and reverse transcription of cDNA was performed using a TUREscript 1st Stand cDNA Synthesis Kit (Aidlab Biotechnologies Co., Ltd., Beijing, China). Primers were designed using Beacon designer 7.9 software for qRT-PCR, and primers are listed in Table 3. qRT-PCR assays were performed on an Analytik Jena-qTOWER 2.2 (Germany) with 2 × SYBR® Green SuperMix (DF, China) and amplified with 1 μL of cDNA template, 5 μL of 2 × SYBR Green Super Mix, and 0.5 μL of each primer to a final volume of 10 μL with water. The amplification programme consisted of one cycle at 95 °C for 3 min, followed by 59 cycles of 95 °C for 30 s and 60 °C for 30 s. The relative expression levels were calculated by the 2^—△△CT^ method [37].

**Table 3:** The primers used for qRT-PCR in this study

No.	Gene ID	Code	Forward primer (F)	Reverse primer (R)
1	TRINITY_DN25157_c0_g1	PGR5	AGGGCACAACCCATGATGAA	TTCGGCTCTTAGACAAAGGCAA
2	TRINITY_DN31568_c1_g5	CHLH	AGATGAGCCAGTTGAACAGAA	AGTAGGAGCCTGAAGCATTG
3	TRINITY_DN27059_c1_g3	BBX24	CAATAGCCTCTCTGCCAACCA	GGTGGCTCTACGCTACTTGTT
4	TRINITY_DN30314_c0_g1	STN7	CCAGATGGGTTACTCGGCTAA	GACTTCTTCTTAGGCTTCGTTTCC
5	TRINITY_DN30720_c0_g10	EXPA8	GGGTCTTGTTACGAAATGCGATGT	CAGAAGTTGGTGGCGGTGAC
6	TRINITY_DN32852_c1_g1	LRR-RLK	GGACCGAGACCTCAATGCTAAGA	TCCTGGTGCTAATGTGAGTGTTCT
7	TRINITY_DN29285_c0_g3	CIPK12	CGAAGAACAACCCGCTCCTCCTC	CGCCCTCGTCGGTCTTGATGT
8	TRINITY_DN31926_c0_g6	bZIP60	TCTTCGTCGTCGTCGTCATC	TTCATCAGCATGTCCTCAACCT
9	Actin		CCTCTATGCCAGTGGTCGTACAA	GCCAGGTCAAGTCGCAGAATG
10	18 s		CAACCATAAACGATGCCGA	AGCCTTGCGACCATACTCC

## Supplementary Information

**Additional file 1: Table S1.** Overview of the sequencing and assembly of the transcriptome.

**Additional file 2: Table S2.** N50 of transcripts or unigenes from nine samples.

**Additional file 3: Table S3.** Species distribution frequency of the BLAST hits based on Nr database.

**Additional file 4: Table S4.** GO classification of the assembled unigenes in *R. xanthina* f. *spontanea. (XLSX 7387 kb)*

**Additional file 5: Table S5.** KEGG function classification the assembled unigenes in *R. xanthina* f. s*pontanea. (XLSX 2156 kb)*

**Additional file 6: Table S6.** GO enrichment analysis of DEGs at 4°C vs. 23°C in *R. xanthina* f. s*pontanea. (XLSX 212 kb)*

**Additional file 7: Table S7.** GO enrichment analysis of DEGs at -20°C vs. 23°C in *R. xanthina* f. s*pontanea. (XLSX 117 kb)*

**Additional file 8: Table S8.** KEGG enrichment analysis of DEGs at 4°C vs. 23°C in *R. xanthina* f. s*pontanea. (XLSX 743 kb)*

**Additional file 9: Table S9.** KEGG enrichment analysis of DEGs at -20°C vs. 23°C in *R. xanthina* f. s*pontanea. (XLSX 318 kb)*

**Additional file 10: Table S10.** GO enrichment analysis of 468 DEGs under low-temperature stress in *R. xanthina* f. *spontanea. (XLSX 91 kb)*

**Additional file 11: Table S11.** KEGG enrichment analysis of 468 DEGs under low-temperature stress in *R. xanthina* f. *spontanea. (XLSX 66 kb)*

**Additional file 12: Table S12.** qRT-PCR analysis of expression levels under low-temperature stress in *R. xanthina* f. *spontanea. (XLSX 16 kb)*

**Additional file 13: Figure S1.** Species distribution of the BLAST hits for each unigenes based on Nr database.

**Additional file 14: Figure S2**. GO enrichment analysis of the assembled unigenes. (DOCX 63 kb)

**Additional file 15: Figure S3.** KEGG pathway functional category of the assembled unigenes. (DOCX 99 kb)

**Additional file 16: Figure S4.** The BUSCO assessment results.

## Data Availability

All data generated in this study are included with its supplementary material. The datasets during the study are deposited in NCBI Sequence Read Archive (SRA) (http://www.ncbi.nlm.nih. gov/ sra /), the accession number is: PRJNA724822.

## Abbreviations

DEGs: Differentially Expressed Genes
KEGG: Kyoto Encyclopedia of Genes and Genomes
qRT-PCR: Quantitative Real-time PCR
GO: Gene Ontology
