# Correction to: Transcriptome and de novo analysis of *Rosa xanthina* f. *spontanea* in response to cold stress

**DOI:** 10.1186/s12870-021-03338-2

**Published:** 2021-12-30

**Authors:** Defeng Zhuang, Ce Ma, Li Xue, Zhen Li, Cheng Wang, Jiajun Lei, Xingfu Yuan

**Affiliations:** 1 Liaoning Academy of Agricultural Sciences, Shenyang, 110161 Liaoning China; 2 Agricultural College, Inner Mongolia Minzu University, Tongliao, 028000 China; 3 College of Horticulture, Shenyang Agricultural University, Shenyang, 110866 Liaoning China; 4 College of Life Sciences and Food Engineering, Inner Mongolia Minzu University, Tongliao, 028000 China

**Correction to: BMC Plant Biol 21, 472 (2021)**

**https://doi.org/10.1186/s12870-021-03246-5**

Following publication of the original article [1], due to typesetting problem, Figs. 1 and 2 were published by mistakes. Therefore, Figs. 1 and 2 have been corrected and listed as follows. I am sorry for the inconvenience.

**Fig. 1:**
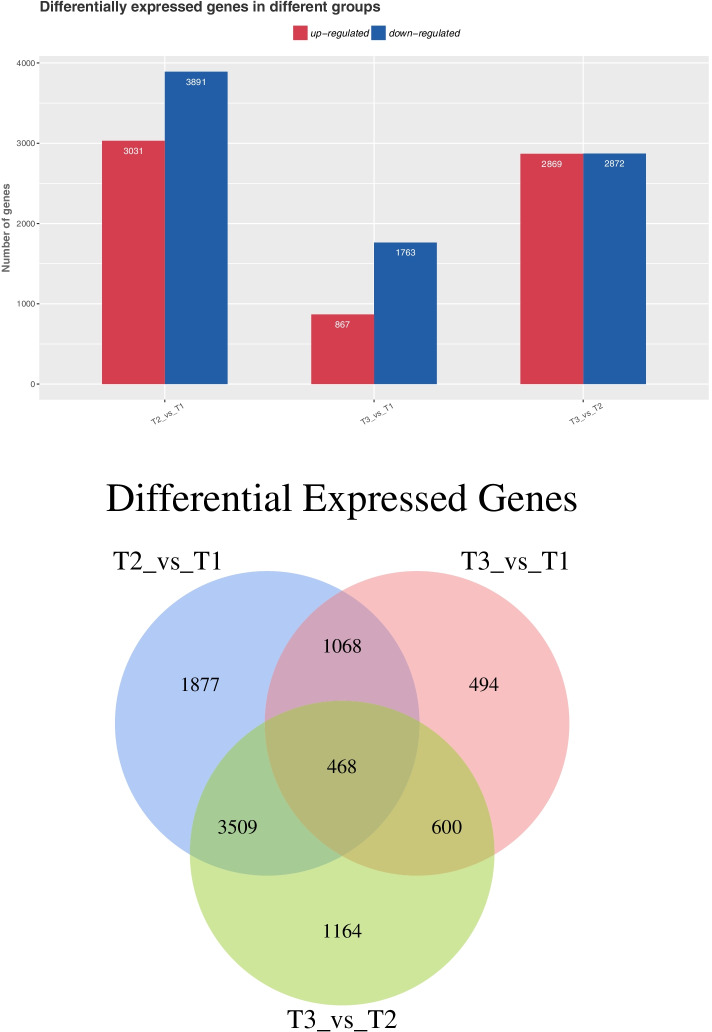
The column and Venn diagrams of DEGs assembled under low-temperature stress across three sets of comparisons expressed as 4 °C vs 23 °C (control), − 20 °C vs 23 °C and 4 °C vs − 20 °C, respectively (*P* < 0.05)

**Fig. 2:**
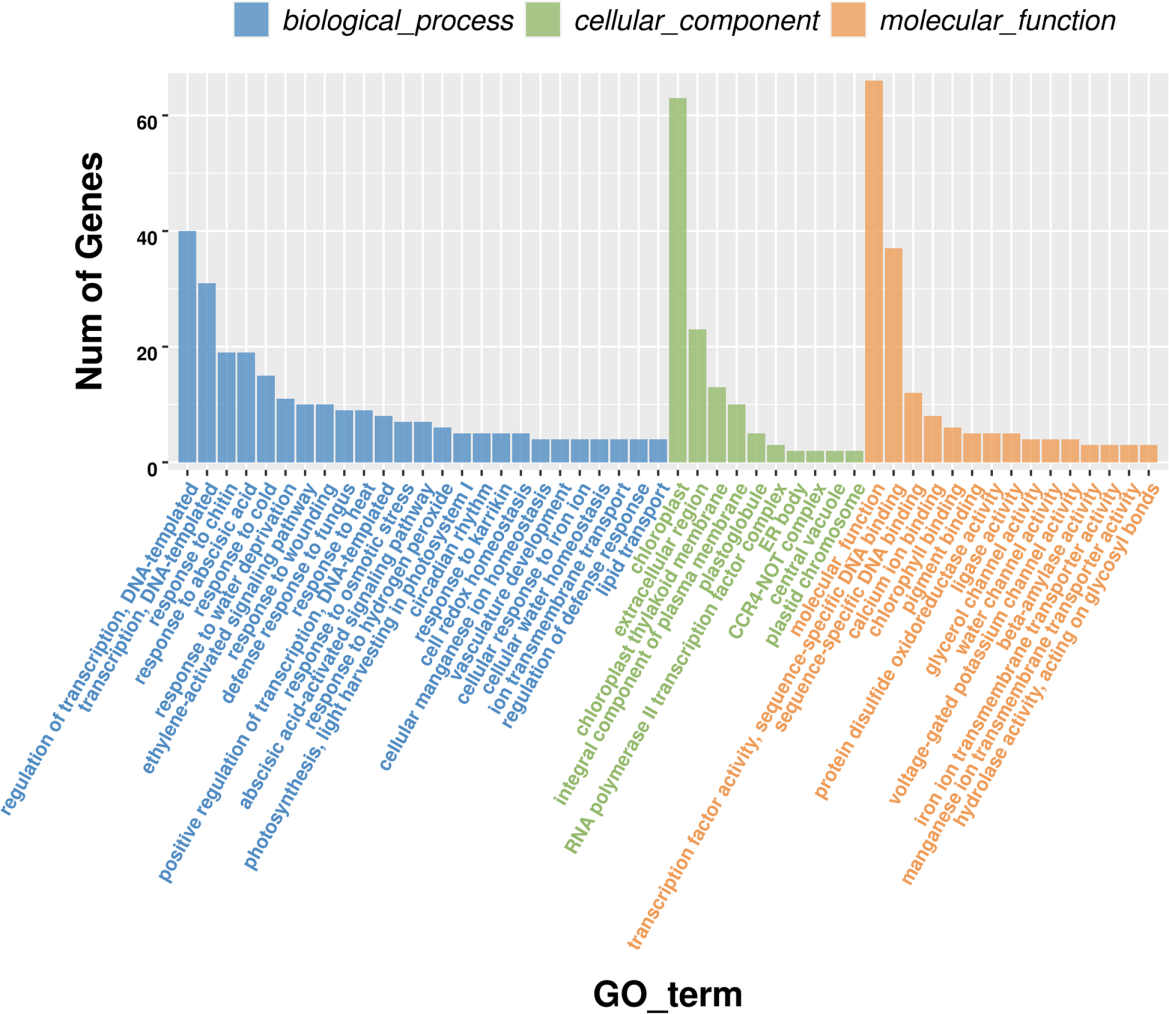
GO enrichment analysis of 468 DEGs. The unigenes were classified into three main categories: biological process, cellular component and molecular function

The correction does not have any effect on the results or conclusions of the paper. The original article has been corrected.
