# Supplementary figures and images for: Transcriptome and de novo analysis of Rosa xanthina f. spontanea in response to cold stress
Source: BMC Plant Biol. 2021 Oct 15;21:472. doi: 10.1186/s12870-021-03246-5 (PMC8518255; doi:10.1186/s12870-021-03246-5)

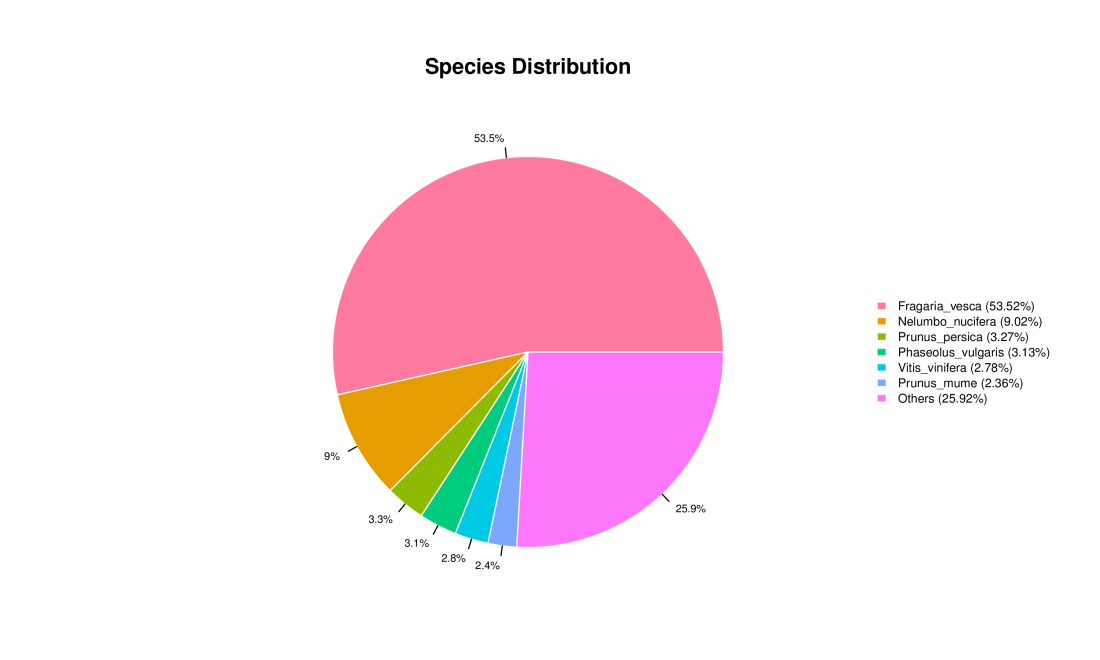


**Fig. S1** Species distribution of the BLAST hits for each unigenes based on Nr database.

Supplement: Supplementary file 13 — Additional file 13: Figure S1. Species distribution of the BLAST hits for each unigenes based on Nr database. [file 12870_2021_3246_MOESM13_ESM.docx]

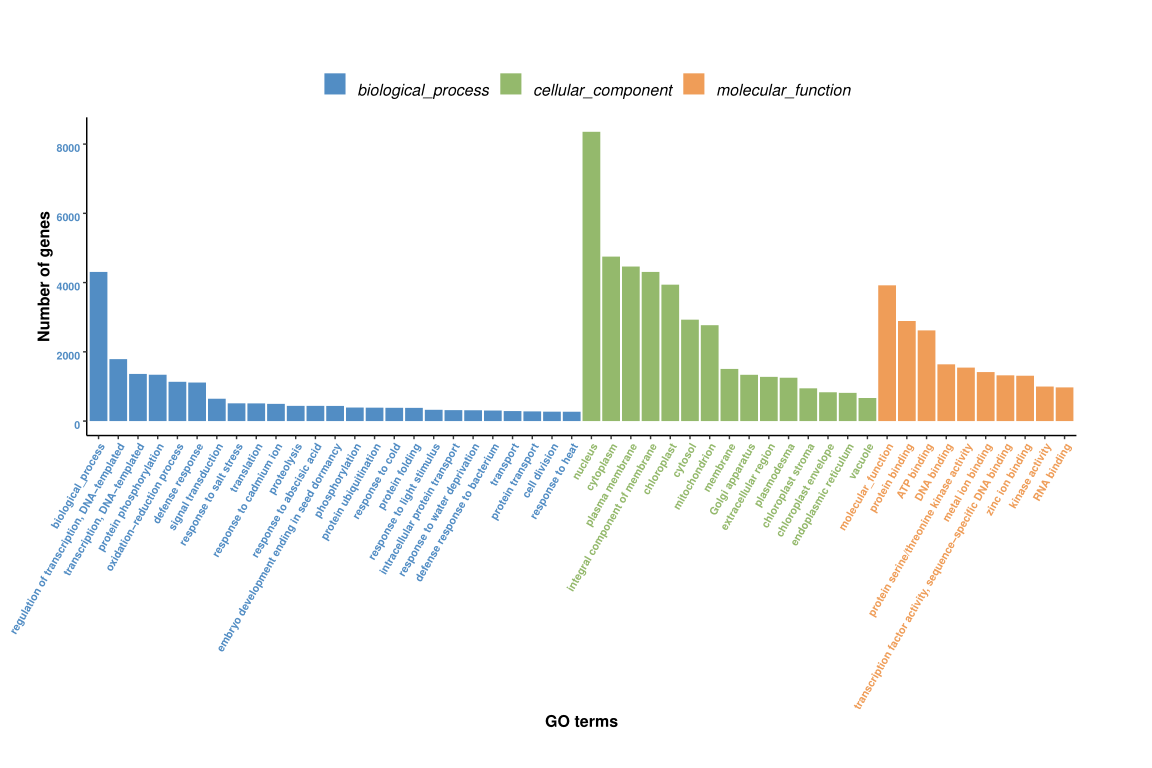


**Fig.S2** GO enrichment analysis of the assembled unigenes.

Supplement: Supplementary file 14 — Additional file 14: Figure S2. GO enrichment analysis of the assembled unigenes. (DOCX 63 kb) [file 12870_2021_3246_MOESM14_ESM.docx]

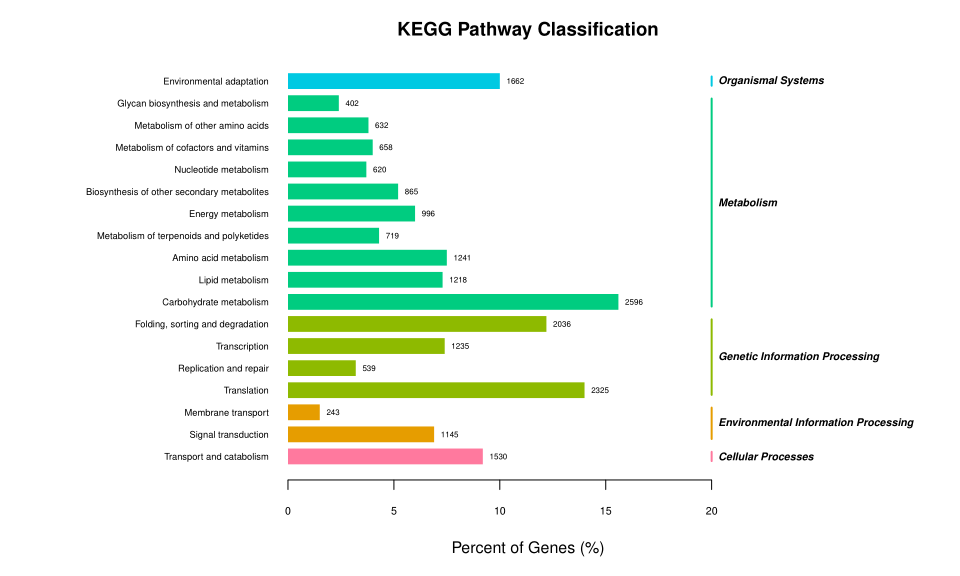


**Fig. S3** KEGG pathway functional category of the assembled unigenes.

Supplement: Supplementary file 15 — Additional file 15: Figure S3. KEGG pathway functional category of the assembled unigenes. (DOCX 99 kb) [file 12870_2021_3246_MOESM15_ESM.docx]

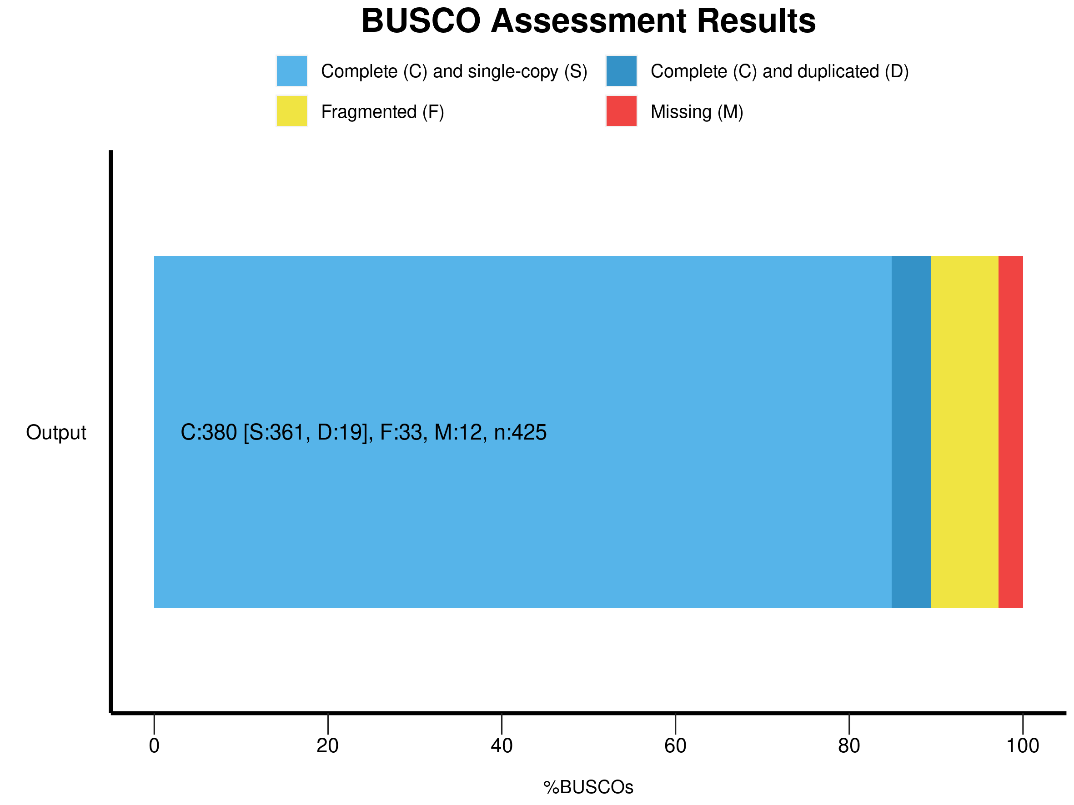


**Fig. S4** The BUSCO assessment results.

Supplement: Supplementary file 16 — Additional file 16: Figure S4. The BUSCO assessment results. [file 12870_2021_3246_MOESM16_ESM.docx]
